# P-1777. AI-Powered Blood Parasite Detection: A Smartphone Diagnostic Tool for Resource-Limited Settings

**DOI:** 10.1093/ofid/ofaf695.1947

**Published:** 2026-01-11

**Authors:** Ryan Vassalotti, Jorge Cervantes, Vanessa D’Amario, Skyler Colwell

**Affiliations:** Dr. Kiran C Patel College of Allopathic Medicine, Plantation, FL; Dr. Kiran C Patel College of Allopathic Medicine, Plantation, FL; Dr. Kiran C Patel College of Osteopathic Medicine, Fort Lauderdale, Florida; Dr. Kiran C Patel College of Allopathic Medicine, Plantation, FL

## Abstract

**Background:**

Blood parasitic infections such as malaria and leishmaniasis continue to pose diagnostic challenges in resource-limited settings, where trained pathologists and appropriate laboratory infrastructure may not be available. Climate change and human displacement through urbanization into sylvatic areas may increase the re-emergence of infectious diseases in what used to be considered remote regions of the world. Furthermore, the risk of coinfection with multiple parasites can further complicate diagnosis, aggravate severity, and pose therapeutic challenges.

**Methods:**

We present an artificial intelligence (AI)-based tool to identify and classify blood parasites from peripheral smear images. Designed for smartphone use, it aims to support accurate diagnostics in remote and low-resource areas.

The AI model was developed using transfer learning with MobileNetV2 as a backbone architecture, allowing for computationally efficient deployment with robust feature extraction. For training, we used a publicly available dataset of annotated microscopy images from Mendeley Data (DOI: 10.17632/38jtn4nzs6.3), containing *Plasmodium, Babesia, Trypanosoma,* and *Leishmania* species as well as leukocytes and RBCs to prevent false positives.

**Results:**

Preliminary results demonstrate an accuracy of 98.24% and an area under the curve (AUC) of 0.9987-0.9999 across the implemented classes on the receiver operating characteristic (ROC) curve when predicting against test data. These results suggest that the model can extrapolate to new data exceptionally well and does not appear to be exhibiting overfitting.

**Conclusion:**

The addition of more blood parasite images could improve prediction accuracy even further. The implementation of the AI model into a smartphone app is underway. In addition, we plan to optimize the cost-effectiveness of this solution by investigating smartphone-attachable microscopes to eliminate the need for expensive microscopy equipment.

AI technology has the potential to significantly improve point-of-care diagnostics in healthcare-underserved communities, especially in remote areas at risk of being affected by the re-emergence of infectious diseases. This technology could support outbreak response and enhance disease surveillance where it is needed most.

**Disclosures:**

All Authors: No reported disclosures

